# Rumination related activity in brain networks mediating attentional switching in euthymic bipolar patients

**DOI:** 10.1186/s40345-018-0137-5

**Published:** 2019-01-12

**Authors:** Kallia Apazoglou, Anne-Lise Küng, Paolo Cordera, Jean-Michel Aubry, Alexandre Dayer, Patrik Vuilleumier, Camille Piguet

**Affiliations:** 1 Department of Psychiatry, Faculty of Medicine, University of Geneva, Geneva, Switzerland; 2 Department of Mental Health and Psychiatry, University Hospital of Geneva, Geneva, Switzerland; 3 Department of Neuroscience, Faculty of Medicine, University of Geneva, Geneva, Switzerland

**Keywords:** Rumination, Bipolar disorder, Self-reference, Attentional switch, sgACC, RRS

## Abstract

**Introduction:**

Mood disorder patients have a tendency to be more internally oriented, with difficulties in switching attentional focus, which might result in the generation of negative thoughts, such as rumination. The present study explored self-referential neural activity correlating with rumination tendency and attentional switching capacity in bipolar disorder.

**Methods:**

Twenty euthymic bipolar patients and twenty matched healthy controls underwent a novel introspection task of switching between internally and externally focused attention during a word processing task, while their brain activity was assessed using functional MRI.

**Results:**

During internal focus, higher activity in self-related regions (mPFC, PCC) was found in euthymic bipolar patients as compared to controls, verifying the hypothesis of exaggerated recruitment of self-referential processes in bipolar subjects. Switching from internal to external focus revealed higher parahippocampal activity in patients as compared to controls, additionally more pronounced when switching away from negative as compared to positive self-referential information. Furthermore, rumination traits correlated with activity in PCC, subgenual and pregenual ACC, and bilateral anterior insula during repetition of internal focus, specifically when evaluating negative words. Finally, we used ACC subregions that correlated with tendency to ruminate as seeds for a whole brain connectivity analysis. Patients showed stronger connectivity between sgACC (seed), pgACC, dPFC, and anterior insula during internal focus, whereas pgACC (seed) was more strongly connected to parahippocampal gyrus when switching from internal to external focus.

**Conclusions:**

These findings reveal an overactive rumination-related network whose activity is enhanced by negative information in euthymic bipolar patients, which could possibly contribute to impaired switching of thoughts away from internal attention.

**Electronic supplementary material:**

The online version of this article (10.1186/s40345-018-0137-5) contains supplementary material, which is available to authorized users.

## Introduction

Mood disorders patients present with deficits in cognitive control and processing biases toward negative material (Gotlib and Joormann 2010). This trait seems to be present both during unipolar and bipolar depression, but also during remitted state for unipolar depression (Gotlib and Joormann 2010) and euthymic phases of bipolar disorder (BD) (Clark and Sahakian 2008). Such impaired cognitive control and negative bias might lead to (1) a difficulty in switching thoughts away from negative material, as demonstrated in unipolar depressive patients (Foland-Ross et al. 2013; Joormann et al. 2011), and (2) a tendency to rumination consisting of intrusive thoughts focused on oneself. Although bipolar and unipolar patients share some common clinical and biological traits (Drevets et al. 2008), a deficit in sustained attention has more specifically been observed in bipolar patients (both depressed and euthymic) but not in unipolar patients (Maalouf et al. 2010). This poor attentional control might alter the ability to direct attention away from distracting stimuli (Maalouf et al. 2010), particularly when these are emotionally relevant (Mullin et al. 2012). Accordingly, recent studies reported that BD patients show deficits in cognitive flexibility in parallel with impairments in sustained attention and information processing for emotionally valenced words (Dickstein et al. 2016).

Rumination is a common feature of mood disorders, characterized by compulsive thinking and excessive focusing of attention on possible causes and consequences of one’s distress (Nolen-Hoeksema et al. 2008). People who tend to ruminate have a higher risk of developing episodes of depression and of relapsing after recovery from depression (Nolen-Hoeksema 2000). Recent hypotheses (Davis and Nolen-Hoeksema 2000; Whitmer and Gotlib 2012) postulate that ruminative thought contents might result from increased limbic activity generating negative affect and negative memories, coupled with diminished prefrontal cortex (PFC) activity responsible for lower executive control and impaired cognitive flexibility (Marchetti et al. 2012). Some neuroimaging data support this model, with trait rumination (measured by the Ruminative Response Scale, RRS) being associated with increased activity in entorhinal cortex (Piguet et al. 2014) or decreased activity in prefrontal areas (Vanderhasselt and De Raedt 2012). Further, induction of rumination may result in increased connectivity within the default mode network (DMN) (Cooney et al. 2010), a set of brain areas linked to self-referential processing, or increased connectivity of the DMN with the subgenual anterior cingulate cortex (sgACC) (Hamilton et al. 2015; Berman et al. 2014). DMN activity during rest is also frequently associated with mind-wandering (Mason et al. 2007), a natural process in the flow of thoughts when participants are not engaged in a task. When associated with negative cognition, mind-wandering has been related to exaggerated self-focus and increased tendency to ruminate as risk factors for mood disorders (Marchetti et al. 2016). Therefore, increased recruitment of the DMN and so-called “self-related” regions, such as mPFC, posterior ACC, and precuneus, is often linked to the frequent occurrence of rumination in mood disorder patients. However, rumination does not only occur during resting state (associated with DMN activity), but intrusive thoughts can also arise during a cognitive task and thus interfere with performance (Piguet et al. 2014). In this case, active rumination might be associated with distinct brain activity patterns, unlike those during unconstrained resting state periods (Berman et al. 2014). Recent imaging work directly comparing brain networks activated during self-reflective processing and resting state, found that, although many regions are similar, the overall pattern of neural activity differs (Davey et al. 2016). Hence, it remains unclear how changes in brain activity associated with ruminations are related to altered cognitive control and impaired attentional flexibility in patients.

In the current study, we designed a novel paradigm that could directly assess brain activity associated with attentional focus on, and away from, self-directed thoughts, and test for their relationship with rumination tendency. While rumination is usually associated with depression or anxiety, there is evidence that bipolar patients also present exaggerated tendency to ruminate, even during euthymic periods (Ghaznavi and Deckersbach 2012; Pavlickova et al. 2013). In addition, impaired cognitive flexibility (Russo et al. 2017) and poor attentional control (Maalouf et al. 2010) across different mood states in BD might contribute to the emergence of intrusive repetitive thoughts in these patients. Based on the above, we hypothesized that BD patients would show (i) increased activity in DMN and related areas, not only as a function of their rumination tendencies, but also (ii) in relation to difficulties in switching attention from an internal to an external focus.

In previous work, internally directed/self-focused processing has been tested by various paradigms ranging from an active induction of rumination (Cooney et al. 2010), through to self-reference tasks (Lemogne et al. 2012) and internally focused meditation (Scheibner et al. 2017). Self-reference processing tasks in mood disorders patients usually imply attributing adjectives to oneself, as compared to others (Lemogne et al. 2012), or manipulating internally as opposed to externally generated information in working memory (Rochat et al. 2012). Here, our novel task aimed to explore more directly the relation of rumination traits with the ability to switch attention from internal to external attention, and to probe this process in a valence-specific context. Negative or positive words were presented each in turn to participants who had to either judge their affective meaning in relation to themselves (i.e., match with current feeling state) or report their external visual features (i.e., number of letters). Critically, we controlled the sequence of word valence and task demands. In line with our second hypothesis, we further expected that euthymic bipolar patients, carrying a vulnerability trait towards more self-focused attention and rumination, would show difficulties in switching attention away specifically from a *negative* internal focus (to an external focus), and that this would be associated with increased activity and/or changes in connectivity of brain areas implicated in self-reflective processes (Davey et al. 2016) (i.e., medial PFC, posterior cingulate cortex—PCC).

## Materials and methods

### Participants and clinical data

Twenty bipolar disorder (BD) patients according to DSM-IV-TR criteria were recruited from the Mood clinic of the Psychiatry Department of Geneva University Hospital, and interviewed by a trained psychologist (PC, ALK) using the DIGS (Diagnostic Interview for Genetic Studies). Patients were included in the study following a 4-week period of euthymic state (defined as Montgomery-Åsberg Depression Rating Scale MADRS level < 13 and Young Mania Rating Scale YMRS level < 6, see Table 1). Both BD type I (n = 11) and II (n = 9) patients were included in the study, with a mean total number of episodes of 8 ± 5. Few comorbidities were diagnosed: 12 patients with substance abuse (mainly alcohol and cannabis), 2 BD patients with obsessive compulsive disorder (OCD), 6 patients with generalized anxiety (GAD), 1 with post-traumatic stress disorder (PTSD) and 7 with attentional deficit and hyperactivity disorder (ADHD). Patients were medicated as follows: 7 with antiepileptic and 5 with antipsychotic drugs, 2 with lithium, 1 with antidepressants and 2 with hypnotic drugs. Twenty healthy controls matched for age, gender, handedness, and educational level (Table 1) were recruited through web announcements and local database. All participants signed a written informed consent (ethical approval from Geneva University CER 13-081).

**Table 1:** Clinical variables: Sample size (n), demographic data and questionnaires scores are given in the table below for both groups as means ±* standard deviation*

	n	Age	Sex	Educational level (years)	YMRS	MADRS	STAI		RRS
							Trait	State	
Bipolar patients	20	33 ±* 10*	10 F 10 M	14 ±* 3.5*	0.85 ± *1.57*	3.31±* 3.45*	47.11 ±* 12.86*	40.29 ±* 14.22*	23.93 ±* 5.3*
Healthy controls	20	32 ±* 10*	10 F 10 M	14.6 ±* 3*	0.68 ± *1.25*	1.21 ±* 1.55*	30.14 ±* 6.98*	26 ±* 4.9*	18.4 ±* 5.16*
p values		*0.74*		*0.57*	*0.72*	***0.02***	< ***0.001***	***0.001***	***0.02***

Statistical* p* values are indicated in italics and significant results (*p* < 0.05) in bold italics

Depression and mania levels were assessed by a trained clinical psychologist (PC, ALK) before the scanning session using the MADRS (Montgomery and Asberg 1979) and YMRS (Young et al. 1978), respectively. Self-report questionnaires were filled in by all participants, covering anxiety (STAI) and tendency to ruminate (Ruminative Response Scale—RRS). We used both versions of the STAI for state and trait anxiety (Bergeron et al. 1976) and a short 10-item version of the RRS (Treynor et al. 2003) comprising only the reflection and brooding subscales.

### Introspection task

**Fig. 1:**
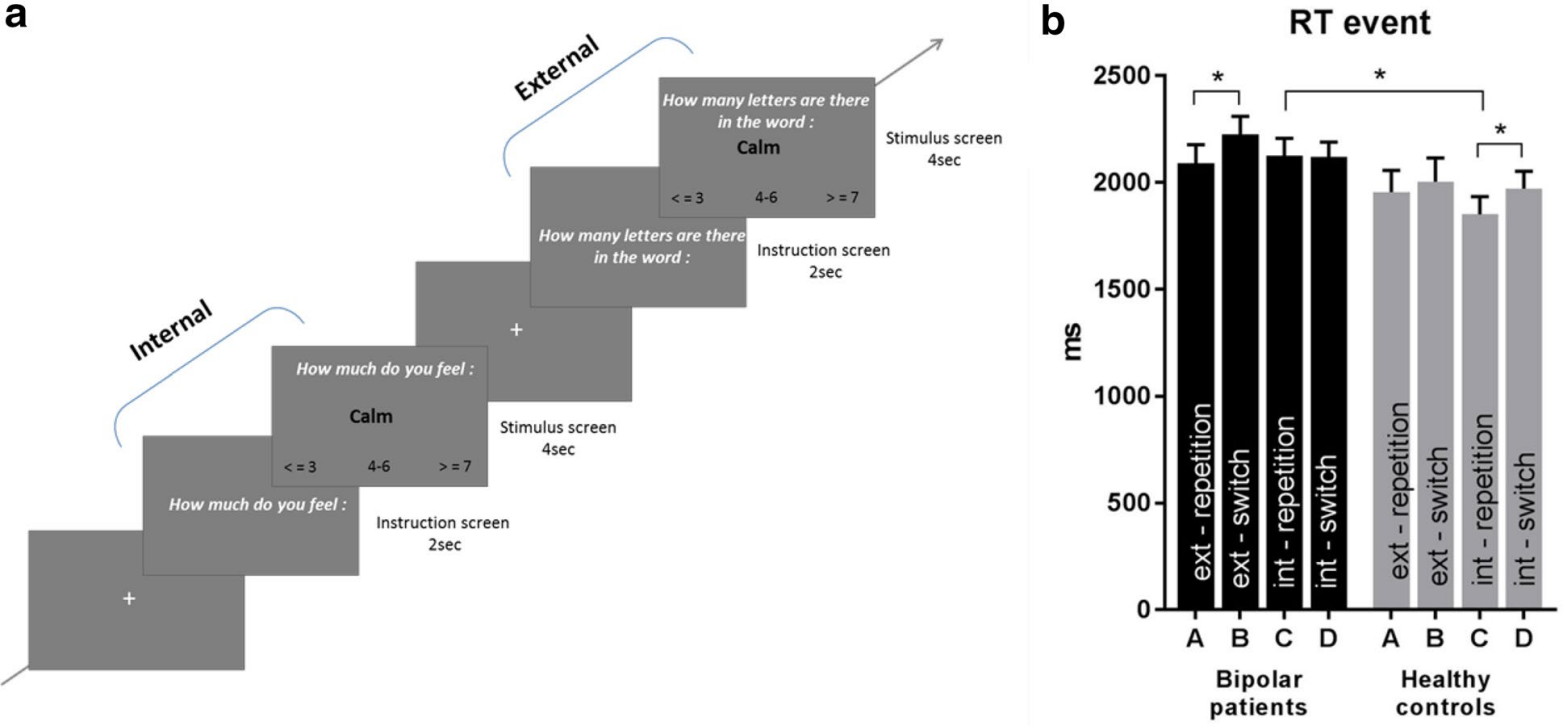
Introspection task and performance. **a** The fMRI task consists of two task conditions: internal and external attentional focus. Each trial begins with an instruction screen displayed during 2 s to indicate the task, followed by a word stimulus with a 3-option rating response displayed for 4 s. During internal trials, participants were asked to evaluate how much the word matched their current internal state. During external trials, they were asked to count how many letters the word included. Word meaning had either a negative or positive valence, and each item was presented in both task conditions. Across successive trials, the task conditions were either repeated or switched, thus yielding a 2 × 2 × 2 design overall l (condition*valence*sequence). **b** Average response times (RT) are shown for each event separately. Patients tend to have slower RTs overall with a marginal main effect of group (0.07), and switch trials were slower than repetition with a significant main effect of sequence (p < 0.01). See text for full statistical analyses of RTs

Each trial started with a 2 s instruction screen, announcing the type of task (internal or external), followed by a 4 s stimulus screen (single word) during which subjects were requested to indicate their response on a 3-point scale (Fig. 1a). Internal trials explicitly asked participants to evaluate how much they were currently feeling the state indicated by the word (an adjective) on a scale with 3 options: low (≤ 3), medium (Joormann et al. 2011; Drevets et al. 2008; Maalouf et al. 2010), high (≥ 7). External trials instead asked participants to indicate the number of letters present in the word on the same scale (< 3, 4–6, 7). The same words and the same three-level scale were used in both tasks, allowing us to equate visual stimulation and cognitive demands on response selection. There were 28 positive and 28 negative items in total, taken from the Profile of Mood State Questionnaire—POMS (McNair et al. 1971), plus 8 clinically relevant items (e.g., “stressed”, “ruminative”). To obtain counterbalanced stimuli for negative and positive conditions, some of the items of the POMS had to be reverted. Each item was presented once in both the internal and external conditions, resulting in 112 trials given in a single session (13 min). Orthogonally to this, words were presented in either the same task (repetition) or different tasks (switch) across successive trials. Valence was equally distributed among switch and repetition trials, and words in switch and repetition trials were randomized and counterbalanced across participants. Mean word length was similar between negative and positive terms. A fixation-cross was shown (jittered duration from 500 to 1500 ms) between successive trials. The task was implemented using E-Prime 2.0 software (Psychology Software Tools Inc., USA), and reaction times and responses were recorded using an MRI-compatible button box (HH—1 × 4—CR, Current Designs Inc., USA). Visual stimuli were displayed using an LCD projector (CP-SX1350, Hitachi, Japan) and projected on a screen at the rear of the scanner, which the participants could comfortably see through a mirror.

### fMRI acquisition

Functional brain images were acquired with a 3T Magnetom TIM Trio scanner (Siemens, Germany) and a 32-channel head coil using a standard echo-planar imaging sequence [36 transverse slices with 20% gap, 64 × 64 base resolution, voxel size: 3.2 mm × 3.2 mm × 3.2 mm, repetition time (TR): 2100 ms, echo time (TE): 30 ms, flip angle (FA): 80°, field of view (FOV): 192 mm]. Image quality was inspected for each participant to ensure the absence of signal drop out in ventral prefrontal regions. Anatomical images were also acquired for precise localization and normalization to standard templates, using a T1-weighted 3D sequence (TR/TI/TE: 1900/900/2.32 ms, flip angle = 9°, field of view = 230 mm, PAT factor = 2, voxel dimensions: 1 mm, isotropic 256 × 256 × 192 voxel). One run of the behavioral task was acquired with 380 scans.

### fMRI data analysis

Image preprocessing and statistical analysis were carried out using standard procedures implemented in SPM12 (http://www.fil.ion.ucl.ac.uk/spm). Functional scans were first realigned using iterative rigid-body transformations that minimize the residual sum of square between the first and subsequent images and corrected for differences in acquisition time between slices. They were then normalized to the MNI EPI template (2D spline, voxel size: 3 mm) and spatially smoothed with a Gaussian kernel with full-width at half maximum (FWHM) of 8 mm. High-resolution structural image was co-registered and normalized with the mean image of the EPI series. Two categorical models (at the individual level) were designed, separating trials according to internal/external focus, sequence of tasks (repetition/switch), and valence. In the first model, the valence of the *current* stimulus was taken into account, in order to investigate the impact of negative vs positive internal focus. In the second model, the valence of the *preceding* stimulus was taken into account in order to study the impact of valence in switching away from negative vs positive internal focus.

1. *Effect of current stimulus valence* 12 regressors of interest (onsets and duration) were defined to model distinct events within each trial as follows: (a) the instruction screen presented at the beginning of each trial (2 s), with 4 different event types based on the task focus (internal or external) and the focus sequence (repetition or switch), leading to 4 conditions: int_int, int_ext, ext_int and ext_ext; (b) the stimulus screen (4 s) with the same 4 event-types and the word valence (positive or negative, 8 conditions in total).
2. *Effect of preceding stimulus valence* In this model the design was similar to the previous (i.e., 12 regressors of interest), but trials were defined according to the valence of the preceding trial and modelled as follows: (a) the instruction screen presented at the beginning of each trial (2 s), with 4 different event types based on the task focus and sequence (internal or external; repetition or switch), and now also the valence of the preceding trial (positive or negative, resulting in 8 event types in total); (b) the stimulus screen (4 s) with 4 event types (internal or external; switch or repetition).

In both models, movement corrections (realignment parameters) were incorporated as covariates (six nuisance regressors). Contrast images were generated for each condition of interest in each participant, and then entered in a second-level (group) analysis using a flexible ANOVA model and random-effects statistics (Penny and Holmes 2004), with depression scores (MADRS) incorporated as a covariate. Main effects are shown at a threshold corrected with the family-wise error fwe p = 0.05, whereas interaction effects are shown at an uncorrected threshold p = 0.001 with a number of voxel > 5 and T score > 3.1.

### Multiple regression

To determine any parametric correlation of brain activity with individual tendency to ruminate, each condition (contrast image) generated for each participant in the first level of analysis was entered in a second level (group) analysis with rumination scores (RRS) as a covariate.

### Psychophysiological interactions (PPI)

To examine task-related modulations of functional connectivity between brain regions according to attention focus and switching conditions, we conducted a PPI analysis using seed regions identified in the group level analysis (BD > HC main effect). The time-courses of selected regions were extracted from the individual (subject) level using the eigenvariate function in SPM. Volumes of interest (VOIs) were then defined as a sphere of 6 mm radius and PPIs computed for each condition. Two analyses were conducted: one using a VOI centred on the subgenual ACC (x = 3, y = 29, z = − 11) and one centred on the pregenual ACC (x = − 6, y = 44, z = 1), as it has been postulated that these regions may be involved in pathological self-referential processing but differentially so (Marusak et al. 2016). A first level analysis (GLM) was conducted using PPIs with psychological estimates for each task condition. Contrasts between conditions were then incorporated in a second level analysis in a flexible factorial statistical design and significant clusters were defined at p = 0.001.

### Mediation analysis

Based on the fMRI results in BD patients, parameters of activity (beta values) from pgACC and entorhinal cortex as well as rumination (RRS) scores were introduced in a mediation analysis. Beta values were extracted from pgACC (x = − 6, y = 44, z = 1) during negative internal repetition trials and from entorhinal cortex (x = − 24, y = − 1, z = − 29) during switch from internal to external as compared to external repetition trials (contrast int.ext > ext.ext), as directed by our hypothesis and our fMRI results. Direct, indirect, and total effects of pgACC (mediator) and entorhinal cortex activities (independent variable) on rumination scores (dependent variable) were concurrently estimated with an implemented script for SPSS software [PROCESS, (Hayes 2017)]. A bootstrap test for the indirect effect (5000 samples, confidence intervals set at 95%) was performed according to Hayes (2017) recommendations.

## Results

### Demographic data

As shown in Table 1, there was no statistical difference between BD patients and healthy controls for age, gender, educational level, and mania scores. Handedness laterality was also matched, with 2 left-handed in the BD group and 3 in the HC group. However, euthymic BD patients scored significantly higher on depression (MADRS, p < 0.05) and anxiety measures (STAI-S, p < 0.01; STAI-T, p < 0.001), and they showed higher tendency to ruminate (RRS, p < 0.05).

### Behavioral data

Response times in the two word-judgment tasks are shown in Fig. 1b. A 2 × 2 × 2 ANOVA showed a marginal group effect (p = 0.07, F = 3.4) and interaction (focus*switch*group, p = 0.055, F = 3.9). Patients were generally slower than controls. Despite the borderline interaction, and given our specific hypothesis concerning the different task conditions, we performed a further analysis of RTs examining internal and external trials separately. A two-way ANOVA (group × switch) on trials from the external focus task testing for the impact of switching *from* the other (internal) task or repeating it showed no group effect (p = 0.18, F = 1.8) but a significant effect of switch (p < 0.05, F = 5.38), and no interaction (p = 0.28, F = 1.18). However, planned comparisons with Bonferroni-corrected t-tests revealed a significant difference between switch and repeat trials in BD patients only (p < 0.05), whereas this difference did not reach significance in HC (p > 0.05). On the other hand, a two-way ANOVA on trials from the internal focus task only showed a marginal group difference (p = 0.05, F = 3.9), with patients being slower than healthy controls, but no other effect. Bonferroni-corrected *t* test comparisons revealed a significant difference of switch vs repeat in HC (p < 0.05) but not in patients, while the difference between groups was significant for repetition trials only (p < 0.05) but not switch trials (p > 0.0.05). Thus, overall, BD patients tended to have more difficulties with the external focus task when it was preceded by internal focus, unlike controls who showed an opposite effect with slower switching from external to internal focus.

### fMRI data

#### Distinct networks activated during internal and external focus

A contrast between all internal vs all external trials (including the instruction and stimulus event periods) across the two groups, at a threshold of p = 0.05 FWE corrected, revealed differential recruitment of widespread brain networks in these two task conditions (see Additional file 1: Figure S1A). In brief, the internal-focus network comprised several midline brain areas usually associated with self-referential processing, including the medial prefrontal cortex, precuneus, and PCC, as well as the lateral orbitofrontal cortex, angular gyrus, middle temporal gyrus, and postero-lateral cerebellum (for detailed coordinates and clusters information see Additional file 1: Table S1A). The external-focus network comprised visual and dorsolateral fronto-parietal areas usually implicated in visuo-spatial attention, as well as the insula cortex and cerebellum vermis (Additional file 1: Table S1B). These results confirm the validity of our task manipulation and its effectiveness in both groups.

#### Main effect of patients vs healthy controls

**Fig. 2:**
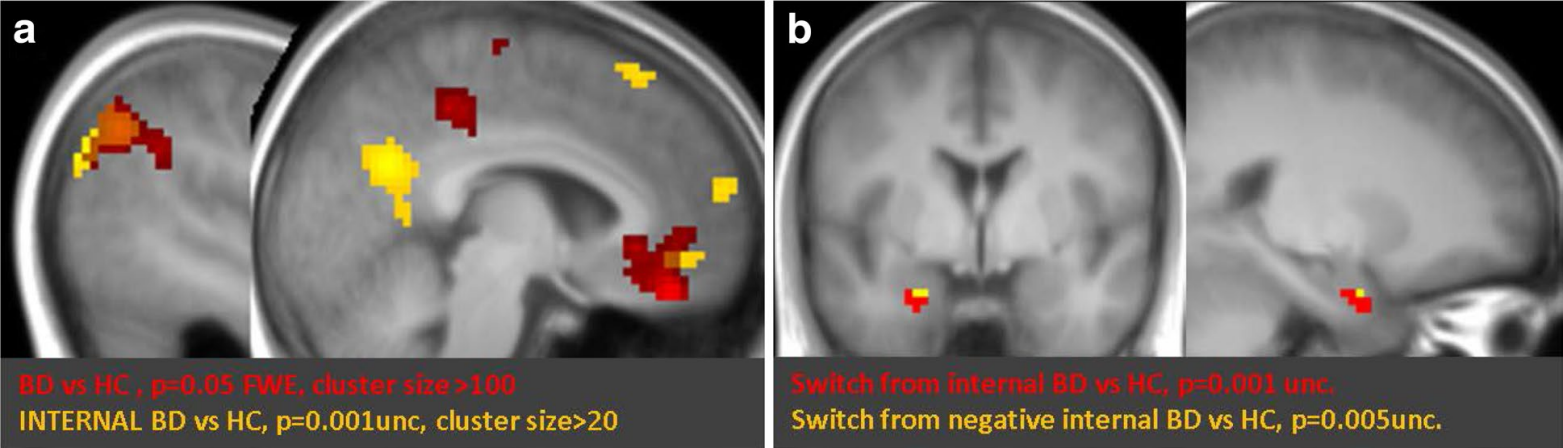
Euthymic Bipolar patients vs healthy controls. **a** The main effect of group (across all conditions) is shown in red at a FWE corrected threshold p = 0.05. Bipolar patients showed higher activity than controls in the vmPFC, PCC, and parietal cortex. The group*attention interaction (BD > HC*internal > external focus) is shown in yellow, revealing clusters associated with internal focus that are also hyperactive in patients. **b** Switching from an internal to an external condition (int.ext) as compared to repeating an external condition (ext.ext) revealed higher activity in the entorhinal cortex for patients as compared to controls (in red, BD > HC*int.ext > ext.ext). The group*valence interaction (BD > HC*int(neg).ext > int(pos).ext) for switching from internal to external focus is shown in yellow, indicating a modulation of switching due to the valence of the previous trial

A direct group comparison across all conditions (BD > HC, p = 0.05 FWE corrected) revealed highly significant increases in the patients for medial brain areas including sgACC (T = 8.08, x = 3, y = 29, z = − 11), vmPFC (T = 9.95, x = − 6, y = 41, z = − 23, T = 8.25, x = − 12, y = 41, z = − 8), and PCC (T = 6.95, x = − 6, y = − 34, z = 46), as well as in the inferior parietal cortex (L: T = 9.10, x = − 45, y = − 61, z = 37, R: T = 8.96, x = 60, y = − 55, z = 28), and superior occipital gyrus (R: T = 13.59, x = 24, y = − 88, z = 34), as shown in Fig. 2a (in red).

To further assess these group differences as a function of task conditions, we computed a group × attention interaction over the whole brain (BD > HC *internal > external, p = 0.001 uncorrected) that revealed higher activity for patients in regions associated with self-referential processing (Fig. 2a, in yellow). These included peaks in the PCC/precuneus (T = 5.19, x = − 3, y = − 61, z = 25), left vmPFC (T = 3.56, x = − 3, y = 44, z = − 11) and dmPFC (T = 3.88, x = − 6, y = 62, z = 16), plus the left middle frontal gyrus (T = 4.95, x = − 30, y = 26, z = 49) and left inferior parietal cortex (T = 4.99, x = − 45, y = − 67, z = 40). These data accord with our hypothesis of differential engagement of self-related processes in patients.

### Switching from internal to external attention

The main effect of switching attentional focus (switch > repetition trials, p = 0.05 FWE, across both groups) showed activity in posterior medial parietal areas, including PCC (T = 8.04, x = 0, y = − 28, z = 28) and precuneus (T = 7.81, x = − 6, y = − 73, z = 40) (Additional file 1: Table S1C), in line with previous research on task switching (Piguet et al. 2016; Yin et al. 2015). Switching from internal to external focus revealed increases in several limbic structures such as amygdala, hippocampus, and striatum across groups, suggesting a delayed deactivation of these areas after a self-reference state (Additional file 1: Table S1D). The reverse contrast showed no effect, i.e., no such inertia of activity in external-focus network.

To test our second hypothesis that patients may have selective difficulties disengaging from internal focus on self-related information, we then compared brain activity between groups on trials that required switching from internal to external focus (*int.ext* condition) as compared to repeated external focus trials (*ext.ext* condition; Fig. 2b, in red). Remarkably, patients (vs controls) showed significantly higher activity in the left entorhinal cortex (T = 4.16, x = − 24, y = − 1, z = − 29), an area that has been previously related to rumination tendency in healthy subjects (Piguet et al. 2014). A further analysis separated trials according to the valence of the preceding word meaning (see “Methods”), in order to compare switching from a *negative* internal trial vs switching from a *positive* internal trial in patients vs controls (interaction of group × valence on *int.ext* trials). Results (Fig. 2b, in yellow) confirmed higher activity in the entorhinal cortex (T = 2.75, x = − 21, y = − 4, z = − 29) in this condition. No such effects were observed when switching from an external to internal attentional focus (i.e., *ext.int* trials) or when repeating the internal focus condition (i.e., *int.int* trials).

### Rumination-related activity in patients

**Fig. 3:**
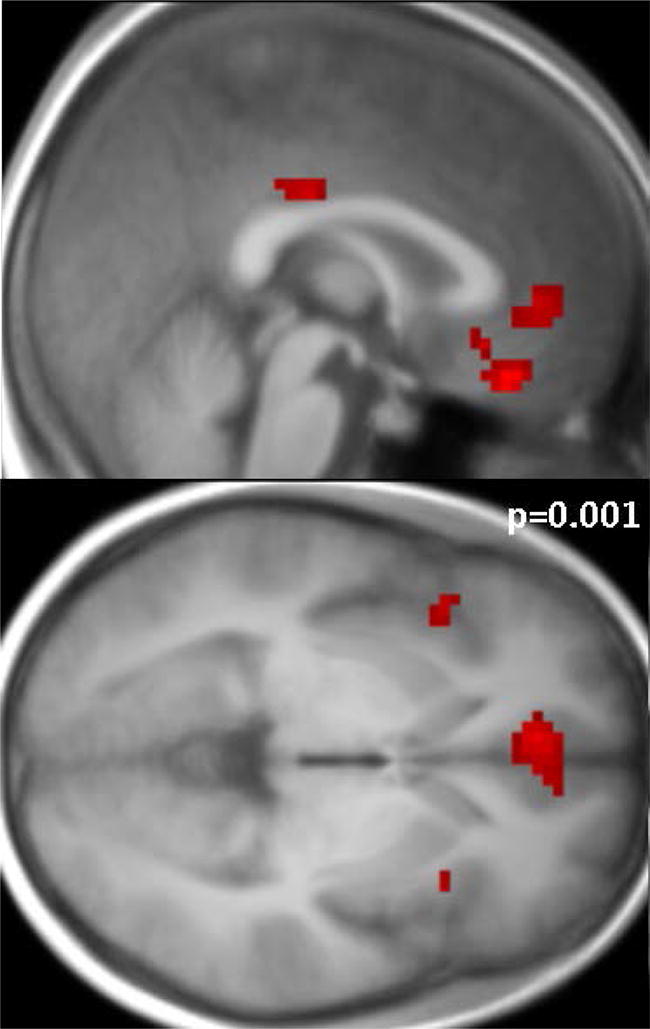
Rumination-related activity in patients. Correlation of tendency to ruminate with brain activity during repeated trials in the internal focus condition (int.int), while patients evaluate their internal state in relation to a word with negative valence. Significant clusters (p = 0.001) were observed in ACC (sub and pre-genual), bilateral anterior Insula, vmPFC, as well as PCC

Next, we performed a whole brain multiple regression analysis using the RRS scores from each individual as a regressor in order to identify areas whose activity increased as a function of ruminative tendencies. Significant clusters (p = 0.001 unc., cluster size > 5) were found during repetition trials specifically for the internal focus condition with negative valence for the patients only. Higher rumination trait was associated with higher activity in this condition in the sgACC (x = 3, y = 29, z = − 11), vmPFC (x = 0, y = 35, z = − 20), pgACC (x = − 6, y = 44, z = 1), bilateral anterior insula (L: x = − 42, y = 17, z = − 2, R: x = 33, y = 17, z = 1), middle/posterior cingulate (x = 3, y = − 25, z = 34), and angular gyrus in parietal cortex (x = − 51, y = − 67, z = 40), as shown in Fig. 3. No significant correlation was found during the external focus repetition or the internal switch condition.

An additional correlation analysis of activity (betas) in clusters that were found more active in BD patients vs HC (i.e. Fig. 2a) was also performed (see Additional file 1: Table S2) and confirmed the specificity of this correlation during negative internal repetition trials. No correlation was observed in any condition in healthy controls. Trait rumination thus appears related to activity in brain areas classically implicated in self-referential processing and self-monitoring, in the condition with the most loading on internal focus.

### Task-specific modulation of functional connectivity of ACC

**Fig. 4:**
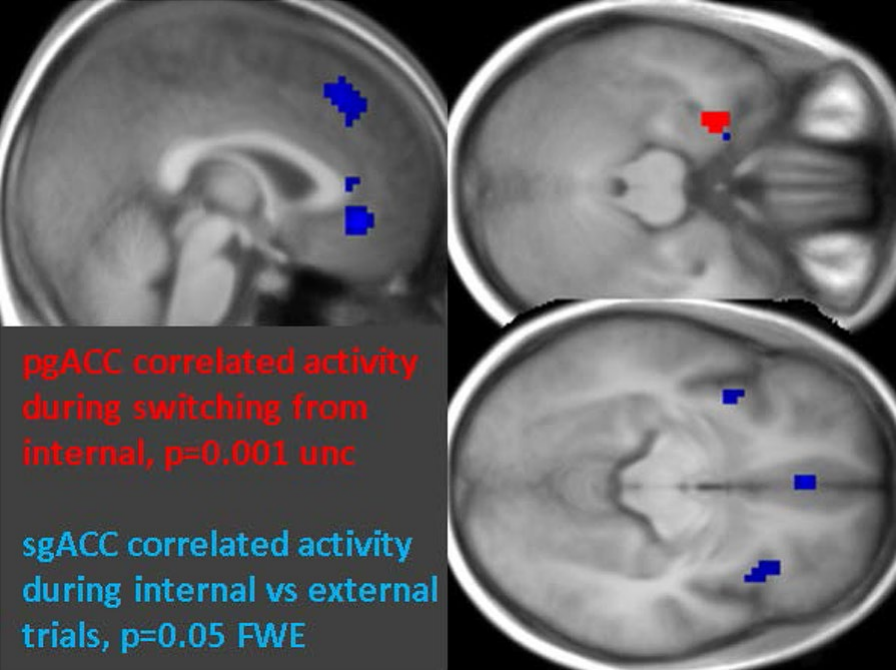
ACC connectivity in patients during internal focus and switch. Psycho-physiological interactions (PPI) in BD patients showing functional connectivity (i) between sgACC (seed region) and areas shown in blue (p = 0.05 FWE) during the internal as compared to the external focus condition; and (ii) between pgACC (seed region) and areas shown in red (p = 0.001 unc.) during attentional switching from an internal to an external focus relative to a repetition of external focus

Finally, we compared functional connectivity of sgACC (x = 3, y = 29, z = − 11) during the internal vs external focus condition in patients using a PPI analysis, since this region is among those hypothesized as central in rumination processes (Hamilton et al. 2015) and depressive state (Mayberg et al. 2005) and was found overactive in our patients as compared to controls. This analysis revealed significantly increased connectivity (p = 0.05 FWE) of sgACC with pgACC (x = − 3, y = 38, z = − 8), medial superior frontal gyrus (x = 0, y = 32, z = 43), and bilateral anterior insula (left: x = − 36, y = 8, z = − 11, right: x = 39, y = 17, z = − 11) (Fig. 4 in blue). A similar analysis also compared connectivity of pgACC when switching away from internal to external focus (vs repetition of external trials) and again found significant increases in patients (p = 0.001 unc, cluster size > 15) centered on the entorhinal cortex (x = − 27, y = − 1, z = − 26) (Fig. 4 in red). Using the same two seeds, no other significant connectivity pattern was found in patients or controls in other conditions. These connectivity results demonstrate increased engagement of self-processing networks in patients, specifically during internal focus, and lingering effects of brain activity related to internal focus when switching to the external focus task.

### Entorhinal cortex activity predicts rumination scores via pgACC activity

**Fig. 5:**
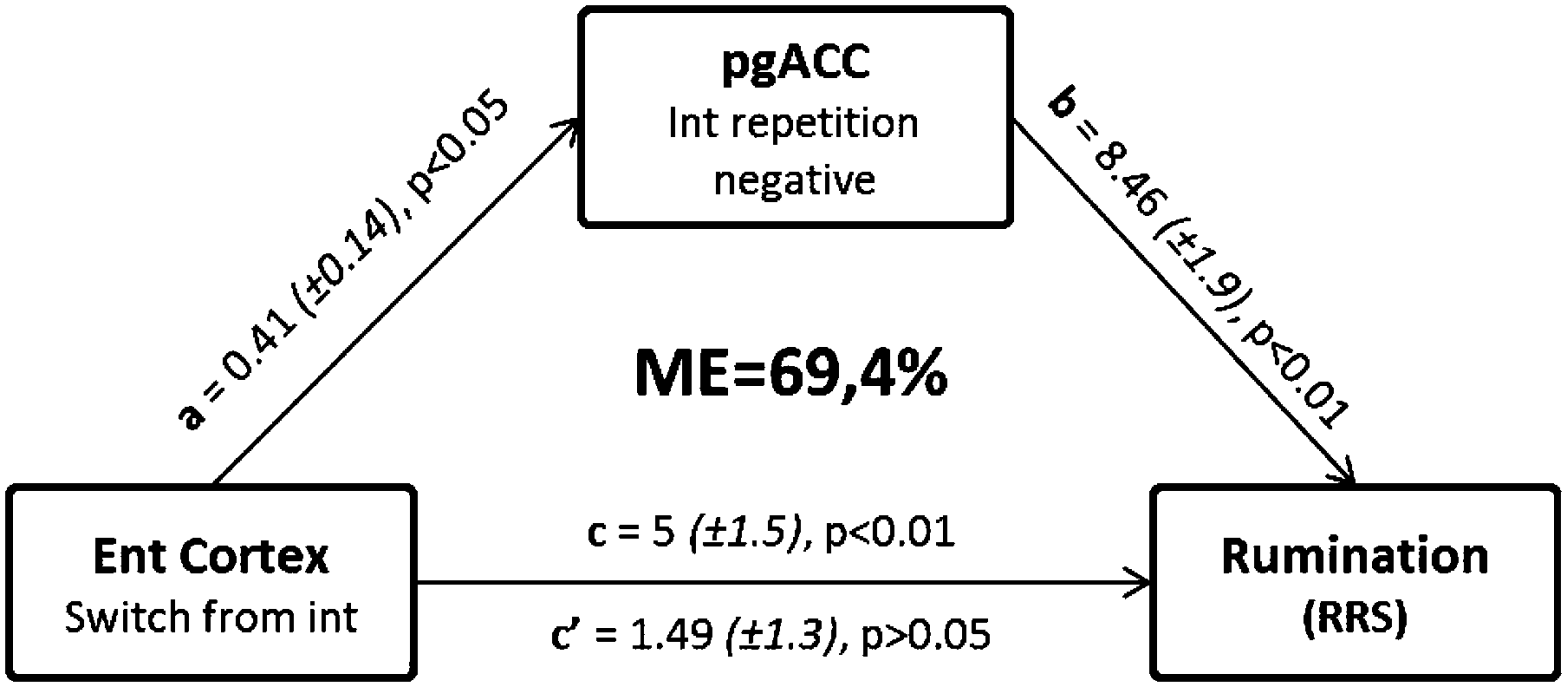
pgACC mediates the link of rumination and entorhinal cortex. Mediation analysis of entorhinal cortex activity (independent variable, X) on rumination trait (dependent variable, Y) via the activity of pgACC (mediator, M). Unstandarized coefficients, standard errors and statistical p values are shown on the arrows indicating paths a (direct effect), b (direct effect), c (total effect) and c’ (indirect effect). Mediation effect (ME = a*b/c) indicates the percentage of the mediator (pgACC) effect to the total effect of entorhinal cortex-induced rumination

A mediation analysis was next conducted, to assess the mediation effect via the pgACC activity of the ability to switch from an internal focus (entorhinal cortex hyperactivity) on rumination trait (RRS scores). In this analysis we defined as “path a” the link between entorhinal cortex and pgACC, as “path b” the link between pgACC and rumination and as “path c” the link between entorhinal cortex and rumination. Mediation analysis from the bootstrap analysis showed a significant indirect effect (M = 3.5, SE = 1.4), with a 95% bias corrected confidence interval excluding zero (1.04, 6.69) and a mediation effect at 69.4%, indicating that the association between the entorhinal cortex activity and rumination passes through pgACC activity. The direct effect and the other coefficient paths of the model were also significant (see Fig. 5 for paths coefficients and p values).

## Discussion

In this study, we designed a novel paradigm to explore brain activity during internal (self-centered) and external (stimulus-centered) focusing of attention in the presence of positive and negative information, and to probe for the effect of switching between these conditions. Euthymic bipolar patients, as compared to healthy controls, showed hyperactivity in midline brain areas associated with self-reflective processing, including vmPFC and both anterior and posterior cingulate cortices. Such increases were observed across all conditions, but more specifically present during internal focus. Moreover, when switching away from internal focus, especially following negative stimuli, patients showed hyperactivity in the entorhinal cortex unlike healthy controls. In addition, connectivity analyses showed that these regions were functionally more coupled together in these conditions, thus highlighting a neural network that may underlie self-directed repetitive thinking in BD. Accordingly, in these patients, trait rumination scores (RRS) were found to correlate with activity in vmPFC, ACC (sub- and pre-genual), anterior insula, and PCC during internal focus, again encompassing regions associated with self-reflective processing. Finally, a mediation analysis showed that trait rumination, if not directly associated with activity in entorhinal cortex, is indirectly so through activity of pgACC. This is the first study, to our knowledge, to elucidate the neural underpinning of the tendency to ruminate and the difficulties in switching attention in relation to exaggerated self-centered activity in euthymic bipolar patients.

### Internally focused attention and rumination-related activity

Behaviorally, BD patients showed an overall tendency to respond slower than controls, but particularly during internal trials. They were also slower in switching from an internal to an external focus as compared to repeating an external trial, and slower when repeating the internal focus condition. These data together suggest a difficulty to disengage from the internal focus or a transient decline in attention due to cognitive or affective overload during self-referential processing. These findings accord with psychopathological models suggesting that the tendency to ruminate reflects or results in executive control deficits in BD (Ghaznavi and Deckersbach 2012) and that a dysfunctional cognitive style with exaggerated internal-focus may impair transitions to a task-positive network (external focus) in remitted depressed patients (Marchetti et al. 2012).

In line with behavioral performance, brain activity during internal focus showed higher recruitment of midline cortical areas in patients, particularly in the ventro-medial PFC and PCC/precuneus, as well as in the parietal angular gyrus. These cortical midline structures overlap with the DMN and have been extensively related to self-referential functions in healthy humans (Northoff et al. 2006), including autobiographical memory (Summerfield et al. 2009) and mind-wandering (Mason et al. 2007). In a recent fMRI study directly comparing self-reference to resting state, Davey and colleagues showed that these areas (mPFC, PCC/precuneus, and left IPL) specifically activate during self-referential mental activity (Davey et al. 2016) and not only during resting state, with self-related content being possibly generated by PCC/precuneus activity and regulated by mPFC. However, self-focused attention during meditation (mindfulness) was recently shown to decrease activity in PCC and medial PFC as compared to mind-wandering in healthy subjects, supporting the hypothesis that exaggerated DMN-like activity may occur during unconstrained trains of thought (Scheibner et al. 2017). Therefore, the respective roles of mPFC and PCC in self-directed thinking (e.g., internally-focused tasks) versus unconstrained mind-wandering or resting state are still debated. Here we show that activity in this network is related to both an exaggerated internal focus and a tendency to ruminate in patients, not to unconstrained thought processes.

Indeed, activity in both anterior (ACC and mPFC) and posterior (PCC) midline regions was correlated with individual rumination scores, specifically during the repetition of negative internal trials. Both sgACC and mPFC have been previously associated to rumination in depressed (Cooney et al. 2010; Johnson et al. 2009) and healthy subjects (Kross et al. 2009). Taken together, these data suggest that ruminative thinking in mood disorder patients might reflect exaggerated and prolonged activity in these areas, triggered by negative self-reference.

In addition, we found that the tendency to ruminate also correlated with activity in bilateral anterior insula (AI). Although less frequently emphasized, a few previous studies suggested a role for AI in self-reflection (Herwig et al. 2012; Modinos et al. 2009), as well as in the integration of self-related information during decisions about mental effort investment (Otto et al. 2014), and in autobiographical self-relevant memories (Araujo et al. 2015). In a recent study investigating episodic counterfactual thoughts, a process similar to rumination where people imagine alternative ways in which past events could have happened, De Brigard et al. (2017) found increased insula activity, together with ACC, medial PFC, and inferior parietal cortex (De Brigard et al. 2017), consistent with our results. Insula has also been associated with heightened interoception in mood disorders (Paulus and Stein 2010). An increased focus on negative interoceptive information might therefore also promote automatic self-directed thoughts focused on past events, and/or contribute to negative affect and anxiety (Knutson et al. 2014) that are associated with self-reference and rumination in patients.

In summary, activity in self-referential brain networks is not only higher in patients as compared to controls, but further directly correlates with tendency to ruminate during negative internal focus, pointing to a plausible neural substrate of self-directed ruminative thoughts. Furthermore, rumination-related activity in regions associated with saliency and interoception, such as the subgenual ACC and bilateral AI, could possibly reflect the implication of these processes in triggering rumination and other affective symptoms of mood disorders, such as anxiety. We further tested how these regions interact as a network.

### ACC connectivity in bipolar patients

Among areas found to be hyperactive and associated with rumination in our BD patients, the sgACC has been consistently implicated in the regulation of automatic emotional behavior (Ghaznavi and Deckersbach 2012; Phillips et al. 2008) and considered as a key node in the neurocircuitry of mood disorders (Drevets et al. 2008; Price and Drevets 2010). Activity in sgACC is increased in both MDD and BD (Piguet et al. 2016), even when corrected for structural differences (Price and Drevets 2010). However, despite these similarities, sgACC blood flow at rest has also been proposed as a specific target discriminating between unipolar and bipolar depression (Almeida et al. 2013).

In our study, activity in sgACC was globally higher in euthymic BD patients compared to healthy controls, correlated with rumination traits during the repetition of internally focused judgments, and showed enhanced connectivity with anterior brain structures (pgACC, mPFC, and insula) during internal focus. Increased functional connectivity of the sgACC with amygdala and PCC has been previously shown by our group in BD patients (relative to healthy controls) during resting state, regardless of their current mood (Rey et al. 2016). Moreover, sgACC-PCC and sgACC-amygdala, as well as sgACC-DMN connectivity has been linked to rumination in other non-euthymic populations (Hamilton et al. 2015; Rey et al. 2016). According to Hamilton et al. (2015), this increased connectivity may represent an integration of self-referential processes (implicating the DMN) with affective information (mediated by the sgACC), forming a circuit substrate for rumination in MDD. Our new data are partially consistent with this hypothesis and therefore point to shared mechanisms for both MDD and BD. Accordingly, as already noted, bipolar patients tend to ruminate equally to MDD patients (Ghaznavi and Deckersbach 2012). Here, when assessing task-related connectivity of the sgACC in BD patients, significant correlations of activity occurred with pgACC, bilateral anterior insula, and dorsomedial PFC during internal focus as compared to external focus, delineating an interconnected network for emotional self-reflection in BD that appears similar to the one expected in unipolar patients. Furthermore, activity in ACC and AI correlated both with the tendency to ruminate and between them, suggesting increased activity in the salience network (Menon and Uddin 2010; Medford and Critchley 2010), as also shown in response to negative-valenced stimuli in MDD (Hamilton et al. 2012). Taken together, our data therefore suggest that sgACC is not only hyperactive in BD patients but also more strongly coupled with pgACC, dorsomedial PFC, and insula during internal focus, with this activity pattern being correlated with the tendency to ruminate when internally focused judgments are repeated and negatively valenced. We might then conclude that such an activity pattern may constitute a dimension shared across mood disorders, in line with a recent meta-analytic study (Marusak et al. 2016) suggesting that both the sgACC and pgACC provide transdiagnostic neural markers reflecting common neurobiological substrates involved in developmental risk (sgACC) or in the broad expression of emotional psychopathology (pgACC) across disease boundaries.

### Switching from internal states—pregenual ACC and entorhinal cortex

While both the sgACC and pgACC were more activated in patients and correlated with rumination traits, we found differential task-related connectivity for these two areas. Unlike sgACC (see above), the pgACC exhibited greater coupling with the left entorhinal cortex when switching from an internal to an external focus in BD patients. Interestingly, the same area was found hyperactive in BD patients relative to HC when comparing the two groups on switching trials. These findings support our hypothesis of higher limbic activity during a switch from negative self-reference states. Moreover, as the entorhinal is critically implicated in memory formation and retrieval (Piguet et al. 2014), our data point to an important role for enhanced communication of ventral parts of ACC with brain systems mediating autobiographical memory during internally focused attention.

Remarkably, the activity in the entorhinal cortex was previously found by our group to correlate with rumination scores in healthy subjects during both resting state and repetition of an easy visual attention task (Piguet et al. 2014), and in another group of bipolar patients (and their healthy controls) during a more difficult double switching task (unpublished results). In the current study, the entorhinal cortex activity during switching from internal to external attention as compared to external repetition trials was also found to significantly correlate with RRS scores in patients and to distinctively couple with pgACC, adding further support to the role of this region and its functional connectivity in relation with the hyperactive self-referential network in BD. As discussed above, activity in pgACC itself was both increased and correlated with rumination in our patients, underscoring a functional link of the entorhinal cortex with the ruminative circuitry. Our mediation analysis provides further evidence in support to this network, suggesting that entorhinal cortex hyperactivity during switch predicts tendency to ruminate only when mediated by hyperactivity of the pgACC. In accordance with our results, increased connectivity between the entorhinal cortex and sgACC has also been reported in bipolar patients during emotion labeling (Almeida et al. 2009). Moreover, entorhinal cortex together with ACC are parts of circuits implicated in automatic emotion regulation (Phillips et al. 2008; Rive et al. 2013) and were found hyperactive in depressed individuals during rumination (Cooney et al. 2010) and emotion regulation (Rive et al. 2013). Our results therefore suggest again a similar involvement in relation to negative self-reference in BD and MDD. The lack of deactivation of this region during switch from internally to externally attention might represent an excessive allocation of attentional resources to self-directed thoughts, at the expense of cognitive processes (Joormann et al. 2011). Finally, this allows refining the network implicated in rumination, often simplified with ROI analyses restricted to mPFC and PCC in current models (Williams 2016).

### General conclusions and limitations

Although our novel paradigm provides important insights into the neural circuitry underlying focus of attention and activity in self-referential brain networks in euthymic bipolar patients, the current study is not without some limitations. First, all patients had medications, of different types and doses, the impact of which cannot be directly addressed here. Second, the clinical heterogeneity of this group (consisting of both bipolar type I and II), together with its modest size, may limit the generalization of our findings. Finally, we did not include another clinical control group, such as unipolar patients, to assess the specificity of brain activation patterns and their relation to different symptom dimensions of mood disorders. Based on our results, it would be necessary to assess if the mechanisms in action pertain to a dimension common to unipolar and bipolar type II disorders, for example, or are found throughout affective disorders in general.

In conclusion, we delineate a hyperactive functionally interconnected network of medial and anterior brain regions encompassing sgACC and pgACC, mPFC, insula, as well as PCC, whose activity is modulated by self-referential processing demands and underpins the tendency to ruminate in BD patients. This network is further connected to other cortico-limbic structures implicated in autobiographical memory, such as the entorhinal cortex, that shows lingering activity in BD when switching from an internal to an external focus. This persistent activation might not only account for heighted neural activity of the DMN at rest, but also contribute to cognitive difficulties in controlling self-referential thoughts and underlie the pronounced ruminative state often observed in mood disorder patients. These data speak in favor of excessive self-referential processing and rumination as central cognitive and neural features in both bipolar and unipolar patients, arguing for specific rumination-focused therapeutic interventions in mood disorders (Topper et al. 2017).

## Additional file

**Additional file 1.** Additional tables and figures.

## Abbreviations

BD: bipolar disorder
MDD: mayor depressive disorder
HC: healthy controls
ACC: anterior cingulate cortex
sgACC: subgenual anterior cingulate cortex
pgACC: pregenual anterior cingulate cortex
PCC: posterior cingulate cortex
PFC: prefrontal cortex
mPFC: medial prefrontal cortex
vmPFC: ventromedial prefrontal cortex
dPFC: dorsal prefrontal cortex
IPL: inferior parietal lobule
DMN: default mode network
STAI: state-trait anxiety inventory
RRS: Ruminative Response Scale
MADRS: Montgomery-Åsberg Depression Rating Scale
YMRS: Young Mania Rating Scale
PPI: psychophysiological interactions
VOI: volume of interest
FWE: family-wise error

## References

[CR1] Almeida JR, Mechelli A, Hassel S, Versace A, Kupfer DJ, Phillips ML (2009). Abnormally increased effective connectivity between parahippocampal gyrus and ventromedial prefrontal regions during emotion labeling in bipolar disorder. Psychiatry Res.

[CR2] Almeida JR, Mourao-Miranda J, Aizenstein HJ, Versace A, Kozel FA, Lu H (2013). Pattern recognition analysis of anterior cingulate cortex blood flow to classify depression polarity. Br J Psychiatry..

[CR3] Araujo HF, Kaplan J, Damasio H, Damasio A (2015). Neural correlates of different self domains. Brain Behav..

[CR4] Bergeron J, Landry M, Bélanger D (1976). The development and validation of a French form of the State-Trait Anxiety Inventory. Cross-Cultural Anxiety..

[CR5] Berman MG, Misic B, Buschkuehl M, Kross E, Deldin PJ, Peltier S (2014). Does resting-state connectivity reflect depressive rumination? A tale of two analyses. NeuroImage..

[CR6] Clark L, Sahakian BJ (2008). Cognitive neuroscience and brain imaging in bipolar disorder. Dial Clin Neurosci..

[CR7] Cooney RE, Joormann J, Eugene F, Dennis EL, Gotlib IH (2010). Neural correlates of rumination in depression. Cognit Affect Behav Neurosci..

[CR8] Davey CG, Pujol J, Harrison BJ (2016). Mapping the self in the brain’s default mode network. NeuroImage..

[CR9] Davis RN, Nolen-Hoeksema S (2000). Cognitive inflexibility among ruminators and nonruminators. Cognit Ther Res.

[CR10] De Brigard F, Parikh N, Stewart GW, Szpunar KK, Schacter DL (2017). Neural activity associated with repetitive simulation of episodic counterfactual thoughts. Neuropsychologia..

[CR11] Dickstein DP, Axelson D, Weissman AB, Yen S, Hunt JI, Goldstein BI (2016). Cognitive flexibility and performance in children and adolescents with threshold and sub-threshold bipolar disorder. Eur Child Adolesc Psychiatry.

[CR12] Drevets WC, Savitz J, Trimble M (2008). The subgenual anterior cingulate cortex in mood disorders. CNS Spectr.

[CR13] Foland-Ross LC, Hamilton JP, Joormann J, Berman MG, Jonides J, Gotlib IH (2013). The neural basis of difficulties disengaging from negative irrelevant material in major depression. Psychol Sci.

[CR14] Ghaznavi S, Deckersbach T (2012). Rumination in bipolar disorder: evidence for an unquiet mind. Biol Mood Anxiety Disord.

[CR15] Gotlib IH, Joormann J (2010). Cognition and depression: current status and future directions. Ann Rev Clin Psychol..

[CR16] Hamilton JP, Etkin A, Furman DJ, Lemus MG, Johnson RF, Gotlib IH (2012). Functional neuroimaging of major depressive disorder: a meta-analysis and new integration of base line activation and neural response data. Am J Psychiatry..

[CR17] Hamilton JP, Farmer M, Fogelman P, Gotlib IH (2015). Depressive rumination, the default-mode network, and the dark matter of clinical neuroscience. Biol Psychiat.

[CR18] Hayes AF (2017). Introduction to mediation, moderation, and conditional process analysis.

[CR19] Herwig U, Kaffenberger T, Schell C, Jäncke L, Brühl AB (2012). Neural activity associated with self-reflection. BMC Neurosci..

[CR20] Johnson MK, Nolen-Hoeksema S, Mitchell KJ, Levin Y (2009). Medial cortex activity, self-reflection and depression. Soc Cognit Affect Neurosci..

[CR21] Joormann J, Levens SM, Gotlib IH (2011). Sticky thoughts: depression and rumination are associated with difficulties manipulating emotional material in working memory. Psychol Sci.

[CR22] Knutson B, Katovich K, Suri G (2014). Inferring affect from fMRI data. Trends Cognit Sci.

[CR23] Kross E, Davidson M, Weber J, Ochsner K (2009). Coping with emotions past: the neural bases of regulating affect associated with negative autobiographical memories. Biol Psychiat.

[CR24] Lemogne C, Delaveau P, Freton M, Guionnet S, Fossati P (2012). Medial prefrontal cortex and the self in major depression. J Affect Disord.

[CR25] Maalouf FT, Klein C, Clark L, Sahakian BJ, LaBarbara EJ, Versace A (2010). Impaired sustained attention and executive dysfunction: bipolar disorder versus depression-specific markers of affective disorders. Neuropsychologia..

[CR26] Marchetti I, Koster EH, Sonuga-Barke EJ, De Raedt R (2012). The default mode network and recurrent depression: a neurobiological model of cognitive risk factors. Neuropsychol Rev.

[CR27] Marchetti I, Koster EHW, Klinger E, Alloy LB (2016). Spontaneous thought and vulnerability to mood disorders: the dark side of the wandering mind. Clin Psychol Sci.

[CR28] Marusak HA, Thomason ME, Peters C, Zundel C, Elrahal F, Rabinak CA (2016). You say ‘prefrontal cortex’ and I say ‘anterior cingulate’: meta-analysis of spatial overlap in amygdala-to-prefrontal connectivity and internalizing symptomology. Transl Psychiatry..

[CR29] Mason MF, Norton MI, Van Horn JD, Wegner DM, Grafton ST, Macrae CN (2007). Wandering minds: the default network and stimulus-independent thought. Science (New York, NY)..

[CR30] Mayberg HS, Lozano AM, Voon V, McNeely HE, Seminowicz D, Hamani C (2005). Deep brain stimulation for treatment-resistant depression. Neuron.

[CR31] McNair DM, Lorr M, Droppleman LF (1971). Educational, Service IT.

[CR32] Medford N, Critchley HD (2010). Conjoint activity of anterior insular and anterior cingulate cortex: awareness and response. Brain Struct Funct.

[CR33] Menon V, Uddin LQ (2010). Saliency, switching, attention and control: a network model of insula function. Brain structure & function..

[CR34] Modinos G, Ormel J, Aleman A (2009). Activation of anterior insula during self-reflection. PLoS ONE.

[CR35] Montgomery SA, Asberg M (1979). A new depression scale designed to be sensitive to change. Br J Psychiatry..

[CR36] Mullin BC, Perlman SB, Versace A, de Almeida JR, Labarbara EJ, Klein C (2012). An fMRI study of attentional control in the context of emotional distracters in euthymic adults with bipolar disorder. Psychiatry Res.

[CR37] Nolen-Hoeksema S (2000). The role of rumination in depressive disorders and mixed anxiety/depressive symptoms. J Abnorm Psychol.

[CR38] Nolen-Hoeksema S, Wisco BE, Lyubomirsky S (2008). Rethinking rumination. Perspect Psychol Sci..

[CR39] Northoff G, Heinzel A, de Greck M, Bermpohl F, Dobrowolny H, Panksepp J (2006). Self-referential processing in our brain—a meta-analysis of imaging studies on the self. NeuroImage..

[CR40] Otto T, Zijlstra FRH, Goebel R (2014). Neural correlates of mental effort evaluation—involvement of structures related to self-awareness. Soc Cognit Affect Neurosci.

[CR41] Paulus MP, Stein MB (2010). Interoception in anxiety and depression. Brain Struct Funct..

[CR42] Pavlickova H, Varese F, Smith A, Myin-Germeys I, Turnbull OH, Emsley R (2013). The dynamics of mood and coping in bipolar disorder: longitudinal investigations of the inter-relationship between affect, self-esteem and response styles. PLoS ONE.

[CR43] Penny W, Holmes AP, Frackowiak RSJ, Penny WD, Zeki S (2004). Random effects analysis. Human brain function.

[CR44] Phillips ML, Ladouceur CD, Drevets WC (2008). A neural model of voluntary and automatic emotion regulation: implications for understanding the pathophysiology and neurodevelopment of bipolar disorder. Mol Psychiatry..

[CR45] Piguet C, Desseilles M, Sterpenich V, Cojan Y, Bertschy G, Vuilleumier P (2014). Neural substrates of rumination tendency in non-depressed individuals. Biol Psychol.

[CR46] Piguet C, Cojan Y, Sterpenich V, Desseilles M, Bertschy G, Vuilleumier P (2016). Alterations in neural systems mediating cognitive flexibility and inhibition in mood disorders. Hum Brain Mapp.

[CR47] Price JL, Drevets WC (2010). Neurocircuitry of mood disorders. Neuropsychopharmacology.

[CR48] Rey G, Piguet C, Benders A, Favre S, Eickhoff SB, Aubry JM (2016). Resting-state functional connectivity of emotion regulation networks in euthymic and non-euthymic bipolar disorder patients. Eur Psychiatry..

[CR49] Rive MM, van Rooijen G, Veltman DJ, Phillips ML, Schene AH, Ruhe HG (2013). Neural correlates of dysfunctional emotion regulation in major depressive disorder. A systematic review of neuroimaging studies. Neurosci Biobehav Rev..

[CR50] Rochat L, Billieux J, Van der Linden M (2012). Difficulties in disengaging attentional resources from self-generated thoughts moderate the link between dysphoria and maladaptive self-referential thinking. Cogn Emot.

[CR51] Russo M, Van Rheenen TE, Shanahan M, Mahon K, Perez-Rodriguez MM, Cuesta-Diaz A (2017). Neurocognitive subtypes in patients with bipolar disorder and their unaffected siblings. Psychol Med.

[CR52] Scheibner HJ, Bogler C, Gleich T, Haynes JD, Bermpohl F (2017). Internal and external attention and the default mode network. NeuroImage..

[CR53] Summerfield JJ, Hassabis D, Maguire EA (2009). Cortical midline involvement in autobiographical memory. NeuroImage..

[CR54] Topper M, Emmelkamp PM, Watkins E, Ehring T (2017). Prevention of anxiety disorders and depression by targeting excessive worry and rumination in adolescents and young adults: a randomized controlled trial. Behav Res Ther.

[CR55] Treynor W, Gonzalez R, Nolen-Hoeksema S (2003). Rumination reconsidered: a psychometric analysis. Cognit Ther Res..

[CR56] Vanderhasselt MA, De Raedt R (2012). How ruminative thinking styles lead to dysfunctional cognitions: evidence from a mediation model. J Behav Ther Exp Psychiatry.

[CR57] Whitmer AJ, Gotlib IH (2012). Switching and backward inhibition in major depressive disorder: the role of rumination. J Abnorm Psychol.

[CR58] Williams LM (2016). Precision psychiatry: a neural circuit taxonomy for depression and anxiety. Lancet Psychiatry..

[CR59] Yin S, Wang T, Pan W, Liu Y, Chen A (2015). Task-switching cost and intrinsic functional connectivity in the human brain: toward understanding individual differences in cognitive flexibility. PLoS ONE.

[CR60] Young RC, Biggs JT, Ziegler VE, Meyer DA (1978). A rating scale for mania: reliability, validity and sensitivity. Br J Psychiatry..

