# Supplementary material for: Rumination related activity in brain networks mediating attentional switching in euthymic bipolar patients
Source: Int J Bipolar Disord. 2019 Jan 12;7:3. doi: 10.1186/s40345-018-0137-5 (PMC6330377; doi:10.1186/s40345-018-0137-5)
Supplement: Supplementary file 1 — Additional file 1. Additional tables and figures. [file 40345_2018_137_MOESM1_ESM.docx]

**Rumination related activity in brain networks mediating attentional switching in euthymic bipolar patients**

Apazoglou et al.

Additional Data

**Figure S1:** A) During internal trials, participants had to evaluate their internal state based on the current word shown on the screen, using a scale with 3 options: low(<=3), medium(4-6), high(>=7). Results are plotted for negative (-) and positive (+) words, for both groups. A 2-way ANOVA revealed a significant main effect of valence (p<0.001) and a significant interaction of group*valence (p<0.05), but no main effect of group. B) During external trials, participants had to count the letters in the current word. Accuracy is plotted for both valences and both groups. A 2-way ANOVA showed only a significant main effect of valence (p<0.001).





**Figure S2:** The main effect of task , showing brain areas differentially activated on internal vs external trials. This contrast includes both the instruction and stimulus periods, pooled across groups, with a threshold of p=0.05 FWE corrected. The internal-focus network is shown in yellow, comprising medial frontal, lateral orbitofrontal, angular and middle temporal cortices, as well as the precuneus, posterior cingulate, and the cerebellum posterior hemispheres. The external-focus network is shown in cyan, comprising occipital, superior and inferior parietal, middle and inferior frontal cortices, as well as the insula and cerebellum vermis.


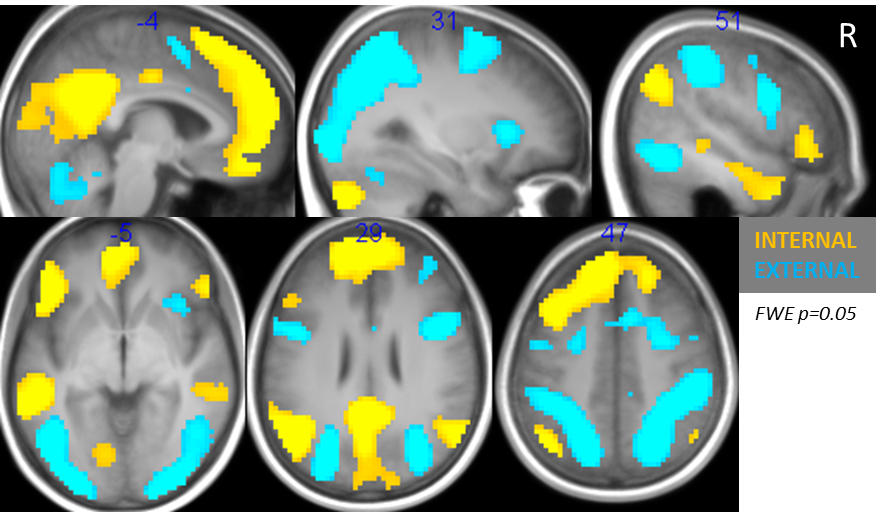


**Table S1 :** Activations observed in the main effects of task.

| 1. **Internal > External p=0.05, fwe** | **x** | **y** | **z** | **Voxels** | **T** |
| --- | --- | --- | --- | --- | --- |
| L Triangularis IFG | *-48* | *29* | *-5* | 5416 | 18.19 |
| L Middle Superior Frontal Gyrus | *-9* | *56* | *25* | 5416 | 18.10 |
| L middle Temporal Gyrus | *-60* | *-43* | *-2* | 5416 | 13.75 |
| L middle Temporal Gyrus | *-57* | *-7* | *-23* | 5416 | 13.13 |
| L Angular Gyrus | *-48* | *-64* | *28* | 5416 | 16.85 |
| L PCC precuneus | *-6* | *-49* | *34* | 1723 | 15.66 |
| L Occipital Pole | *-9* | *-97* | *16* | 1723 | 7.58 |
| R Occipital Pole | *15* | *-94* | *22* | 1723 | 7.23 |
| R cerebellum exterior | *24* | *-85* | *-35* | 392 | 13.26 |
| R cerebellum exterior | *45* | *-61* | *-47* | 392 | 6.56 |
| R angular Gyrus | *57* | *-61* | *31* | 252 | 11.48 |
| R Triangularis IFG | *57* | *32* | *4* | 488 | 10.12 |
| R middle Temporal Gyrus | *60* | *-4* | *-23* | 488 | 8.69 |
| R middle Temporal Gyrus | *54* | *5* | *-32* | 488 | 7.22 |
| L cerebellum exterior | *-27* | *-82* | *-35* | 117 | 9.58 |
| R middle Temporal Gyrus | *63* | *-40* | *-2* | 141 | 7.69 |

| 1. **External > Internal P=0.05, fwe** | **x** | **y** | **z** | **Voxels** | **T** |
| --- | --- | --- | --- | --- | --- |
| R Superior Parietal | *27* | *-67* | *37* | 2452 | 13.59 |
| R Superior Parietal | *27* | *-58* | *58* | 2452 | 13.35 |
| R Inferior Occipital gyrus | *30* | *-94* | *-5* | 2452 | 13.04 |
| L Inferior Occipital gyrus | *-33* | *-94* | *-5* | 2252 | 12.91 |
| L Superior Parietal | *-24* | *-67* | *34* | 2252 | 12.56 |
| L Superior Parietal | *-24* | *-58* | *55* | 2252 | 12.38 |
| R Precentral gyrus | *48* | *5* | *25* | 242 | 12.24 |
| R Precentral gyrus | *48* | *11* | *4* | 242 | 5.37 |
| R Middle Frontal gyrus | *27* | *-1* | *52* | 702 | 9.88 |
| R Middle Frontal gyrus | *30* | *5* | *61* | 702 | 9.63 |
| R Supplementary Motor Cortex | *3* | *5* | *55* | 702 | 6.76 |
| L Middle Frontal gyrus | *-24* | *5* | *55* | 702 | 7.35 |
| L Precentral gyrus | *-45* | *2* | *31* | 183 | 8.88 |
| L Precentral gyrus | *-48* | *-7* | *49* | 183 | 6.31 |
| R anterior Insula | *33* | *20* | *4* | 116 | 8.06 |
| Cerebellum Vermis VI-VII | *-3* | *-73* | *-35* | 280 | 6.67 |
| Cerebellum Vermis VIII-X | *0* | *-64* | *-35* | 280 | 6.64 |
| L Cerebellum Exterior | *-18* | *-70* | *-47* | 280 | 6.40 |
| R Middle Frontal gyrus | *36* | *44* | *28* | 124 | 6.02 |
| R Middle Frontal gyrus | *48* | *41* | *19* | 124 | 5.95 |

| 1. **Switch > repetition, p=0.05 fwe** | **x** | **y** | **z** | **Voxels** | **T** |
| --- | --- | --- | --- | --- | --- |
| Posterior Cingulate gyrus | *0* | *-28* | *28* | 154 | 8.04 |
| L precuneus | *-6* | *-73* | *40* | 410 | 7.81 |
| R precuneus | *9* | *-70* | *40* | 410 | 6.53 |

| 1. **Switch from internal p=0.05, fwe** | **x** | **y** | **z** | **Voxels** | **T** |
| --- | --- | --- | --- | --- | --- |
| R cerebellum exterior | *30* | *-85* | *-29* | 528 | 8.67 |
| R cerebellum exterior | *18* | *-88* | *-29* | 528 | 8.04 |
| R inferior occipital gyrus | *42* | *-82* | *-5* | 528 | 5.15 |
| L occipital fusiform gyrus | *-42* | *-73* | *-17* | 308 | 6.31 |
| L inferior occipital gyrus | *-35* | *-88* | *1* | 308 | 6.05 |
| L middle occipital gyrus | *-33* | *-91* | *13* | 308 | 5.89 |
| L posterior cingulate gyrus | *-3* | *-43* | *10* | 19 | 6.15 |
| L supplementary motor cortex | *-3* | *8* | *70* | 29 | 5.50 |
| R caudate | *9* | *9* | *-3* | 92 | 5.84 |
| R accumbens area | *9* | *8* | *-8* | 92 | 5.70 |
| L accumbens area | *-9* | *5* | *-5* | 54 | 5.87 |
| L hippocampus | *-36* | *-25* | *-14* | 33 | 5.71 |
| L middle cingulate gyrus | *0* | *11* | *34* | 16 | 5.64 |

**Correlations between rumination and brain activity**

To examine whether the observed hyperactivity in patients is related to rumination traits beta values were extracted from clusters identified in the aforementioned comparisons; precisely in the sgACC (x=3, y=29, z=-11), left vmPFC (x=-9, y=50, z=-8) and PCC (x=-6, y=-34, z=46) with coordinates taken from the group comparison main effect (Figure 2B, in red), then in the left superior medial frontal gyrus (x=-6, y=62, z=16), left frontal middle gyrus (x=-30, y=26, z=49), left parietal angular cortex (x=-45, y=-67, z=40) and PCC/precuneus (x=6, y=-58, z=22) with coordinates taken from the interaction with attentional focus (internal vs external, Figure 4, in yellow), and finally in the left entorhinal cortex (x=-24, y=-1, z=-29) with coordinates taken from the switch internal to external focus (Figure 2B). Activation of these areas was then correlated with RRS scores across conditions. Detailed results are shown in Table S2 in supplementary data. Significant positive correlation was found between rumination scores and activity in sgACC (r=0.78, p=0.006), PCC/precuneus (r=0.52, p=0.04), vmPFC (r=0.63, p=0.01) and angular cortex (r=0.65, p=0.009) specifically during a negative internal focus trial and in particular after repetition of internal focus. No correlation was observed in PCC, parahippocampal, frontal superior medial or middle cortices. To note, no correlation was found in any of those clusters when testing for the control group.

**Table S2:** Pearson’s correlations of beta values with RRS during the instruction screen (cue) and the stimulus screen (negative or positive word). The statistically significant ones are shown in red. Beta values were extracted from ROIs (sphere, 3mm diameter) defined in the sgACC (x=3, y=29, z=-11), vmPFC (x=-9, y=50, z=-8), frontal superior medial (x=-6, y=62, z=16), frontal middle (x=-30, y=26, z=49), PCC (x=-6, y=-34, z=46), precuneus (x=6, y=-58, z=22), parietal angular L (x=-45, y=-67, z=40) and parahippocampal cortex L (x=-24, y=-1, z=-29).

|  | **External repetition** | | | **External switch** | | | **Internal repetition** | | | **Internal switch** | | |
| --- | --- | --- | --- | --- | --- | --- | --- | --- | --- | --- | --- | --- |
| **Brain areas** | **cue** | **negW** | **posW** | **cue** | **negW** | **posW** | **cue** | **negW** | **posW** | **cue** | **negW** | **posW** |
| **sgACC** | 0.46 | 0.04 | 0.29 | 0.40 | 0.20 | 0.33 | 0.48 | **0.78** | **0.58** | **0.53** | 0.10 | 0.22 |
| **vmPFC** | 0.00 | -0.24 | -0.27 | -0.26 | -0.02 | -0.10 | -0.08 | **0.68** | 0.48 | 0.01 | -0.17 | -0.21 |
| **medPFC** | -0.04 | -0.24 | 0.07 | 0.17 | -0.26 | 0.13 | 0.14 | 0.44 | -0.01 | -0.17 | 0.26 | 0.00 |
| **midPFC** | -0.07 | -0.42 | -0.09 | -0.22 | -0.23 | -0.11 | -0.11 | 0.31 | -0.13 | -0.40 | 0.14 | -0.14 |
| **L parietal** | 0.44 | -0.19 | -0.06 | 0.18 | 0.03 | 0.19 | 0.24 | **0.69** | 0.50 | -0.03 | **0.60** | 0.52 |
| **PCC** | 0.26 | -0.38 | -0.21 | 0.09 | -0.33 | 0.09 | 0.11 | 0.06 | -0.29 | -0.09 | -0.16 | -0.26 |
| **precuneus** | -0.12 | 0.13 | 0.18 | -0.11 | 0.18 | 0.33 | 0.04 | **0.62** | 0.31 | 0.05 | 0.12 | 0.21 |
| **entorhinal** | 0.20 | -0.07 | -0.11 | -0.01 | 0.13 | 0.27 | 0.15 | -0.05 | -0.08 | 0.19 | -0.13 | -0.20 |
